# Diverse Responses of Oligodendrocytes to Different FGF-Family Members: Uncoupling Structure-Function Relationship Within FGF Subfamilies

**DOI:** 10.1080/17590914.2024.2371163

**Published:** 2024-07-15

**Authors:** Hebe M. Guardiola-Diaz, Brett T. DiBenedictis, Erealda Prendaj, Rashmi Bansal

**Affiliations:** a Department of Biology and Neuroscience Program, Trinity College, Hartford, Connecticut, USA; b Department of Neuroscience, University of Connecticut Medical School, Farmington, Connecticut, USA

**Keywords:** Myelin, myelination, oligodendrocyte, oligodendrocyte progenitors

## Abstract

The fifteen canonical paracrine fibroblast growth factors (FGFs) are organized in five subfamilies that interact with four FGF-receptors (FGFRs) and heparan sulfate proteoglycan (HSPG) co-receptors. Many of these FGFs are expressed in CNS regions where oligodendrocyte (OL) progenitors originate, migrate or differentiate. FGF2 (basic FGF) is considered a prototype FGF and the information about the effects of FGF signaling on OL-lineage cells has evolved largely from the study of FGF2. However, other FGFs from four subfamilies ((FGF1 (FGF1,-2), FGF4 (FGF4,-5,-6), FGF8 (FGF8,-17,-18) and FGF9 (FGF9,-16,-20)) that can interact with the isoforms of FGFRs expressed in OL-lineage cells may also play important roles. We previously reported OL-responses to FGF8 family members. Here, we investigate the effects of members of the FGF1,-4, and -9 subfamilies on proliferation and differentiation of OL progenitors (OPCs), and on cell cycle re-entry and down-regulation of myelin proteins by mature OLs. We found that while FGF2 induced all these responses strongly, FGF4,-6,-9 could do so only transiently and in the presence of exogenous HSPGs, and that FGF5,-16,-20 could not do so even in the presence of heparin or at higher concentrations. Furthermore, we noted that structurally similar FGFs within subfamilies did not always show similarities in their biological effects on OL-lineage cells. Taken together, these studies reveal that FGFs differ in the way they regulate the OL-lineage cells, emphasizes the selectivity and importance of HSPGs as FGF co-receptors in OL-lineage cells and suggests that structural similarity among FGF-subfamily members may not always predict their overlapping biological functions.

## SUMMARY STATEMENT

Structurally similar members of the FGF1, -4, and -9 subfamilies trigger diverse biological responses in oligodendrocyte-lineage cells and exhibit selective requirement for heparan sulfate proteoglycans as FGF co-receptors.

## Introduction

During development, oligodendrocyte (OL) lineage cells transition through defined developmental stages in response to multiple signals that include several growth factor families. These signals play important roles in regulating OL progenitor cell (OPC) proliferative and migratory potential, govern differentiation into mature OLs and regulate myelin formation (Bergles & Richardson, 2015; Clayton & Tesar, 2021; Cristobal & Lee, 2022; Emery, 2010; Miller, 2002). OPCs persist in the adult brain, but they typically fail to differentiate and to efficiently replace damaged myelin in multiple sclerosis and other demyelinating disorders (Sim et al., 2002). Having a better understanding of signaling mechanisms by growth factors may unlock the therapeutic potential of these cells to form new myelin in the adult CNS. The fibroblast growth factor (FGF) signaling system offers particularly promising opportunities to regulate various stages of OL development and myelin formation because of the great combinatorial potential of its multiple ligands, receptors, co-receptors and intracellular signaling effectors.

The FGF family is composed of 22 members, which are divided into 7 subfamilies based on their structural similarities. Out of these, 15 FGFs are secreted canonical paracrine FGFs, 3 are endocrine FGFs and 4 are intracellular proteins (intracellular FGFs or iFGFs) that primarily function to regulate the activity of voltage-gated sodium channels and other molecules in neurons (Xie et al., 2020; Ornitz & Itoh, 2022). The 15 canonical paracrine FGFs are divided into 5 subfamilies. These include: FGF1 subfamily (FGF1, FGF2), FGF4 subfamily (FGF4, FGF5, FGF6), FGF7 subfamily (FGF3, FGF7, FGF10, FGF22), FGF8 subfamily (FGF8, FGF17, FGF18) and FGF9 subfamily (FGF9, FGF16, FGF20). These FGFs signal through the activation of four-tyrosine kinase FGF-receptors (FGFR1-4) and use heparan sulfate proteoglycan molecules (HSPGs) as co-receptors (Allen & Rapraeger, 2003; Li & Kusche-Gullberg, 2016; McKeehan et al., 1998; Ornitz, 2000; Turnbull et al., 2001). These ternary FGF-HSPG-FGFR complexes modulate multiple signaling pathways, including the Ras/Raf/Mek/Erk, PI3K/Akt/mTOR and PLC pathways (Itoh & Ornitz, 2004; Mason, 2007; Turner & Grose, 2010). Thus, the diverse temporal and spatial expression of FGFs, FGFRs and HSPGs confers the capability of regulating multiple responses in target cells.

Many of these FGFs are expressed in the nervous system and regulate key developmental processes (Eckenstein, 1994; Ford-Perriss et al., 2001; Ornitz & Itoh, 2015; Reuss & von Bohlen und Halbach, 2003; Turner & Grose, 2010; Xie et al., 2020). FGF2 is considered the archetypal FGF and more is known about the cellular responses to FGF2 than the other FGF family members. In the CNS, FGF2 is expressed by both neurons and astrocytes, whereas FGF1, the second member in the FGF1 subfamily, is predominantly expressed in neuronal axons, concomitantly with active myelination (Becker-Catania et al., 2011; Elde et al., 1991; Fon Tacer et al., 2010; Gomez-Pinilla et al., 1992; Matsuyama et al., 1992; Ratzka et al., 2011; Riva & Mocchetti, 1991).

We have previously shown that three of the four FGFRs (FGFR1,-2,-3) and a select group of heparan sulfate proteoglycans are expressed by the OL-lineage cells in a developmentally regulated manner (Bansal et al., 1996a, 1996b; Fortin et al., 2005). OPCs express FGFR1 and FGFR3 but not FGFR2 while differentiated OLs express FGFR2 and to a lesser extent, FGFR1 but not FGFR3. In addition, FGFR2, but not FGFR1, continues to be expressed in adult non-compact myelin, enriched in “lipid raft” microdomains (Bryant et al., 2009). In the embryonic ventral forebrain, FGFRs are expressed (Bansal et al., 2003) and orchestrate the generation of OPCs (Furusho et al., 2011) involving the activation of the major docking protein FRS2 (Furusho et al., 2020). Also, conditional ablation of FGFR2 in oligodendrocytes leads to attenuation of myelin growth during the active phase of myelination, demonstrating an important role of FGFR2 signaling in the regulation of myelin assembly in the CNS (Furusho et al., 2012). Furthermore, we have shown that this FGFR2-mediated process involves Erk1/2 and mTORC1 activation (Furusho et al., 2017). More pertinent to this study, we and others have demonstrated *in vitro* that FGF2 stimulated OPC proliferation and inhibited their differentiation (Baron et al., 2000; McKinnon et al., 1990) and that FGF2 added to mature OLs induced re-entry into the cell cycle and down-regulated their myelin proteins, including FGFR2 expression (Bansal & Pfeiffer, 1997; Fressinaud et al., 1995; Fortin et al., 2005). Despite this significant progress in our understanding of the various functions of FGFRs *in vivo* and the multiple response of OL-linage cells to FGF2 *in vitro*, important questions regarding the role of other FGF family members remain unanswered.

Out of the 15 canonical paracrine FGFs from 5 subfamilies, we focused on11 FGFs from 4 subfamilies, namely FGF1 subfamily (FGF1, FGF2), FGF4 subfamily (FGF4, FGF5, FGF6), FGF8 subfamily (FGF8, FGF17, FGF18) and FGF9 subfamily (FGF9, FGF16, FGF20), because they are expressed in the CNS and are capable of interacting with the isoforms of FGFRs expressed by the OL-lineage cells. We previously examined the responses of cultured OPCs and OLs to the 3 members of the FGF8 subfamily (FGF8,-17,-18) and reported that in contrast to the multiple effects of FGF2 described above, these FGFs triggered only a limited number of responses (Fortin et al., 2005).

In this study, we examined the role of 8 additional members of the remaining 3 subfamilies of interest to obtain a more complete understanding of the responses to FGFs and the FGF signaling system in OL-lineage cells. Specifically, we evaluated the responses of OPCs and OLs to subfamilies FGF1 (FGF1, -2), FGF4 (FGF4, -5, -6) and FGF9 (FGF9, -16, -20) relative to responses induced by FGF2. We also examined the requirement of HSPGs in modulating these responses. We found that unlike FGF2, which elicited all responses strongly, the responses to FGF4, -6, -9 were transient and required the presence of exogenous heparin as a co-receptor, while FGF5, -16, -20 could not induce any of the responses even with heparin and at higher concentrations. Thus, this study shows diversity in the way different FGFs affect the OL-lineage cells and revels a selectivity in their requirement of HSPGs as co-receptors. Furthermore, FGFs within structurally similar subfamilies did not always show similarities in their biological effects, suggesting an uncoupling of the structure-function relationship within FGF subfamilies.

## Materials and Methods

### Cell Culture

Purified populations of OPCs and mature OLs were prepared from Sprague Dawley neonatal rats (RRID:RGD_737891). OPC purity and phenotype were characterized by immuno-labeling with a panel of antibodies as described previously (Bansal et al., 1996a; Bansal & Pfeiffer, 1997). Briefly, OPCs were obtained from mixed primary cultures from neonatal rat telencephalon (P1-2) by overnight shaking in an orbital shaker (McCarthy & de Vellis, 1980), followed by differential adhesion to remove astrocytes and macrophages. Complement lysis with anti-galactocerebroside (GalC), that marks mature OLs, was additionally performed for the enrichment of OPCs by removing a few terminally differentiated OLs. OPCs were plated in 5% FCS (fetal calf serum)/DMEM (Dulbecco’s Modified Eagle’s Medium) in tissue culture plates coated with poly-L-ornithine (Sigma, St. Louis, MO; 50 µg/ml). Following cell attachment for 3 hours, the medium was changed to serum-free, defined medium (mN2) [DMEM with human transferrin (50 µg/ml), bovine pancreatic insulin (5 µg/ml), 3,3,5-triiodo-L-thyronine (10 ng/ml), sodium selenium (45 nM), D-biotin (10 ng/ml), hydrocortisone (10 nM), sodium pyruvate (0.11 mg/ml), penicillin-streptomycin (10 IU/ml and 100 µg/ml, respectively)] and 0.05% BSA (all from Sigma)]. PDGF-BB (Upstate Technologies; 10 ng/ml) was added to promote OPC proliferation and kept in the culture until desirable density was achieved while arresting their differentiation into OLs. After adequate PDGF expansion, cultures were medium changed to mN2. For OPC experiments, different FGFs (PeproTech Inc. Rocky Hill, New Jersey) and heparin (10,000 units, #H-3149 Sigma, St Louis, MO) were added to cultures at this point. In OPC experiments lasting more than two days of FGF treatment, we replenish FGFs and heparin on the second day of the experiment with a media change. This FGF exposure time is consistent with the bioactivity testing conducted by PeproTech. For differentiated mature OL experiments, OPCs were left in mN2 to allow them to differentiate into mature OLs and then different FGFs were added, and cells were grown for 2 more days before analysis. For immunoblot analysis of proteins, OL cultures were grown in 35 mm dishes at high density.

FGFs used in this study were: FGF1 (# 100-17 A), -2 (# 100-18B), -4 (# 100-31), -5 (# 100-34), -6 (# 100-30), -9 (# 100-23), -16 (# 100-29) and -20 (# 100-41). These were all human recombinant proteins produced by PeproTech in *E. coli*, purified (>95% purity) and analyzed by SDS PAGE. They were made to contain mature active sequences available at the time of production. A minor revision of the sequences was made since then for FGF6, -19 and -20 in which the N-terminus were extended by 3, 1 and 2 amino acids, respectively. These FGFs were shown to be bioactive in bioassays which demonstrated cellular responses to each individual FGFs. Doses of 10 ng/ml were initially used for all FGFs. Because at this dose we did not observe any response to FGF5, -16 or -20, we used these FGFs at a higher dose to ask whether they might reveal responses not evident at 10 ng/ml. Therefore, for all experiments presented in this paper, FGF1,-2,-4,-6, and -9 were used at 10 ng/ml and FGF5,-16 and -20 were used at 20 ng/ml.

### Immunofluorescence Microscopy

Live cells were immunolabeled on ice as described previously (Bansal et al., 1996a). Briefly, cells were blocked for non-specific absorption with HEPES buffered Earl’s balanced salt solution (EBSS-HEPES) containing 3% normal goat serum (also used for diluting antibodies) and double-immunolabeled with OL-lineage stage-specific markers

OPCs were immunolabelled with monoclonal antibodies A2B5 (hybridoma cells obtained from ATCC Cat# CRL-1520, RRID:CVCL_7946) and O4 (hybridoma cells gift from Dr. M. Schachner, Department of Neurobiology, University of Heidelberg, Germany; Bansal et al., 1992; Sommer & Schachner, 1981). Mature OLs were immunolabeled with an antibody that recognizes HPC, a protein co-expressed with galactocerebroside in differentiated OLs (gift from Dr. C.J. Barnstable, Department of Ophthalmology and Visual Science, Yale University School of Medicine, New Haven, Connecticut; Baas & Barnstable, 1998). Cells were then labeled with the appropriate secondary antibodies [FITC-conjugated goat anti-mouse IgM (µ-chain specific, for O4, and A2B5; Jackson Labs., Bar Harbor, Maine); Cy3-conjugated anti-mouse IgG (gamma-chain specific for HPC; Jackson Labs., Bar Harbor, Maine) and a nuclear label, Hoechst dye (Sigma)]. Between steps, cells were washed 3 times with five-minute exchanges of EBSS-HEPES. Cells were then mounted and examined by epifluorescence microscopy.

To identify proliferating cells in the S-phase of the cell cycle, cultures were exposed to bromodeoxyuridine (BrdU) at a final concentration of 50 µM for 3 h at 37 °C for its incorporation into newly synthesized DNA. Cells were then stained live for A2B5/O4, then fixed with ethanol/glacial acetic acid (95:5) at −20 °C (2 min), denatured with 2 N HCl (10 min), neutralized with 0.1 M sodium borate (pH 8.5; 10 min), incubated with anti-BrdU (Becton-Dickinson, Lincoln Park, NJ; 1:50; 20 min) followed by goat anti-mouse IgG conjugated to Cy3 (Jackson Laboratory) and mounted for immunofluorescent examination and analysis.

### Immunoblotting

Cells grown in 35 mm dishes were harvested in lysis buffer (10 mM Tris-HCl, 150 mM NaCl, 0.1% SDS, 1% deoxycholate, 1% NP40 and 1% TX-100, pH 7.4) with protease and phosphatase inhibitors (1 mM PMSF, 10 µg/ml leupeptin, 10 µg/ml aprotinin, 1 mM orthovanadate, Na fluoride 50 mM, Na pyrophosphate 10 mM) on ice and cup sonicated (30 s; 4°C). The homogenates were then incubated (10 min, on ice) and centrifuged (15,000 × g, 15 min, 4°C). The protein concentration was assayed with the DC Protein Assay Kit (Bio-Rad, Hercules, CA). Aliquots of equal amounts of total protein from different experimental conditions were electrophoresed on 12% SDS polyacrylamide gels and transferred onto PVDF membranes. The membranes were blocked for 30 min (Tris buffered saline, 0.2% Tween 20, and 5% non-fat powdered milk or 5% BSA), incubated for 1 hr in primary antibodies: myelin oligodendrocyte glycoprotein (MOG; gift from Dr. C. Linington, Aberdeen, UK); FGFR2, (Santa Cruz Biotech, CA, Cat# sc-6930, RRID:AB_669015); β-actin (Sigma, St Louis, MO); p21cip1 (BD Biosciences, San Jose, CA BD Biosciences Cat# 556431, RRID:AB_396415); mCNP (BioLegend, Lutherville, MD Cat# SMI 91, RRID:AB_2565362). The membranes were then incubated for 30 min in appropriate secondary antibodies conjugated to horseradish peroxidase (1:10,000; Santa Cruz). The membranes were developed using the ECL Plus kit (Amersham, Arlington Heights, IL).

### Statistical Analysis

Data are presented as mean ± SEM. All statistical analyses were performed using Student’s paired t tests with a critical probability of *p* < .05. Standard errors of the mean were calculated for all data using N values from multiple independent experiments (stated in figure legends) each performed in triplicate wells. Cell counts were performed from at least 10 fields of view per well using 40× magnification. For proliferation and differentiation studies: approximately 200–500 cells were counted/well.

## Results

Our previous studies have suggested that while FGF2 induced multiple, developmental stage-specific responses in OL-lineage cells, FGF8 subfamily members (FGF8,-17,-18) could only trigger a subset of these responses (Fortin et al., 2005). Here we investigated the cellular responses of OL-lineage cells to the remaining FGF subfamily members that are known to be expressed in the nervous system and activate FGFR in the OL-lineage cells. These eight FGFs are members of subfamilies FGF1 (FGF1,-2), FGF4 (FGF4,-5,-6) and FGF9 (FGF9,-6,-20). Briefly, we treated OPCs and mature OLs in culture with these FGFs and examined their effects on cell proliferation and differentiation in the absence or presence of exogenously added heparin (a pan-HSPG). Immunolabelling of cells with developmental stage-specific antibodies, in conjunction to cell morphology, was used to identify cells within specific stages of development as described previously (Bansal et al., 1996a; Pfeiffer et al., 1993). In brief, A2B5 marks only early OPCs, O4 marks both late OPCs and differentiated OLs, whereas HPC marks only differentiated OLs. Therefore, total OL-lineage cells at a given time in culture are identified as A2B5+/O4+ cells and mature OLs as HPC + cells.

Proliferation of OPCs and cell cycle reentry by mature OLs were determined by BrdU immunolabeling. As an additional indicator of proliferation, we examined the expression of p21cip1. This is because in our previous studies (Bansal et al., 2005) we had found that FGF2 treatment, which caused an increase in OPC proliferation, as evaluated by increased BrdU incorporation and increased OPC cell counts, also caused an increase in the level of p21cip1. We had described this finding as paradoxical because p21cip1 is generally considered as an inhibitor of cell cycle progression known to contribute to G1 checkpoint control to prevent S-phase entry. However, we showed that elevated levels of p21cip1 by FGF2 treatment can positively regulate cell cycle progression in OPCs depending upon the temporal expression of other regulators of cell cycle which we had also determined in this study.

### Diverse Effects of FGFs on the Proliferation of Oligodendrocyte Progenitors

**Figure 1.**
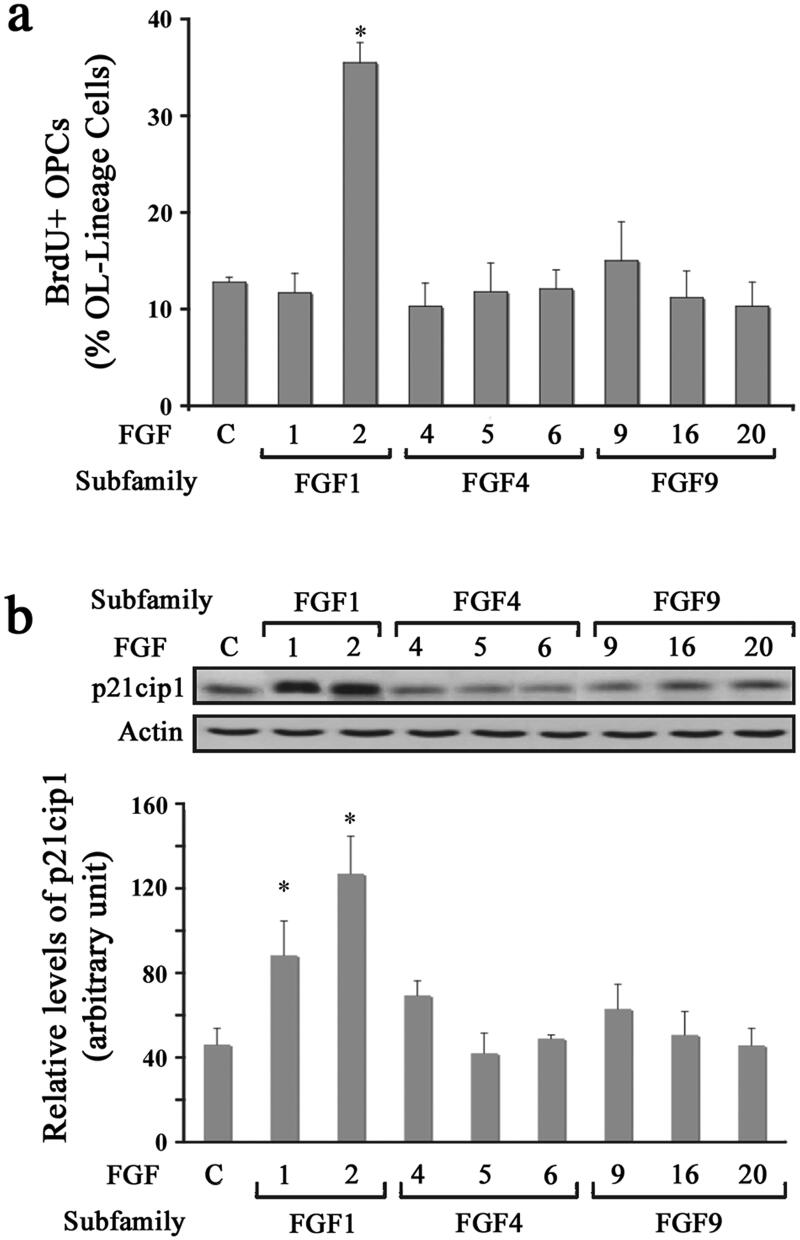
Diverse effects of FGFs on the proliferation of oligodendrocyte progenitors. OPCs were grown in the absence (control) or presence of FGF1, FGF2, FGF4, FGF5, FGF6, FGF9, FGF16, or FGF20 for 1–2 d and their effect on proliferation was analyzed. (a) The quantification of the numbers of BrdU + OPCs, expressed as percentage of total OL-lineage cells (A2B5+/O4+ cells) show that only FGF2 induces a statistically significant increase in proliferating OPCs compared with controls. (b) Quantification of the levels of p21cip1 after 2 d of exposure to FGFs show that FGF1 and FGF2 caused a statistically significant increase in its levels. Error bars represent SEM; *N* = 3 independent experiments, each performed in triplicate. *p < .05. FGF1, FGF2, FGF4, FGF6, FGF9 were used at 10 ng/ml and FGF5, FGF16, FGF20 at 20 ng/ml. Inset, representative immunoblot for the expression of p21cip1 and actin as protein loading control.

To determine the effect of different FGFs on the proliferation of OPCs, we analyzed BrdU incorporation in response to FGF1, FGF2, FGF4, FGF5, FGF6, FGF9, FGF16, or FGF20 by immunolabelling cells with anti-BrdU following BrdU exposure in culture (Figure 1a). While a base level of untreated OPCs incorporated BrdU (∼10%), a three-fold increase in the percent of BrdU + cells was observed in FGF2 treated cultures. In contrast, treatment with all other FGFs (FGF1,-2,-4,-5,-6,-9,-16 and -20) failed to stimulate BrdU incorporation. We then performed immunoblotting of protein lysates for p21cip1 levels from OPC cultures following a 2-day treatment with different FGFs (Figure 1b). Quantification of signal intensity showed that consistent with BrdU incorporation, there was an increase in the levels of p21cip1 in OPCs treated with FGF2 but not with FGF4, FGF5, FGF6, FGF9, FGF16, or FGF20. Thus, the relationship between BrdU incorporation and p21cip1 levels held true in all the cases except in the case of FGF1, where p21cip1 levels increased in the absence of an increase in BrdU incorporation. The reason for this is not clear. However, since BrdU incorporation is a universally accepted indicator of proliferation, by this established criterion it appears that FGF1 did not induce OPC proliferation. Nevertheless, the functional significance for the FGF1-mediated increase in p21cip1 levels in the absence of an increase in BrdU incorporation remains unanswered at this time.

### Diverse Effects of FGFs on the Differentiation of Oligodendrocyte Progenitors

**Figure 2.**
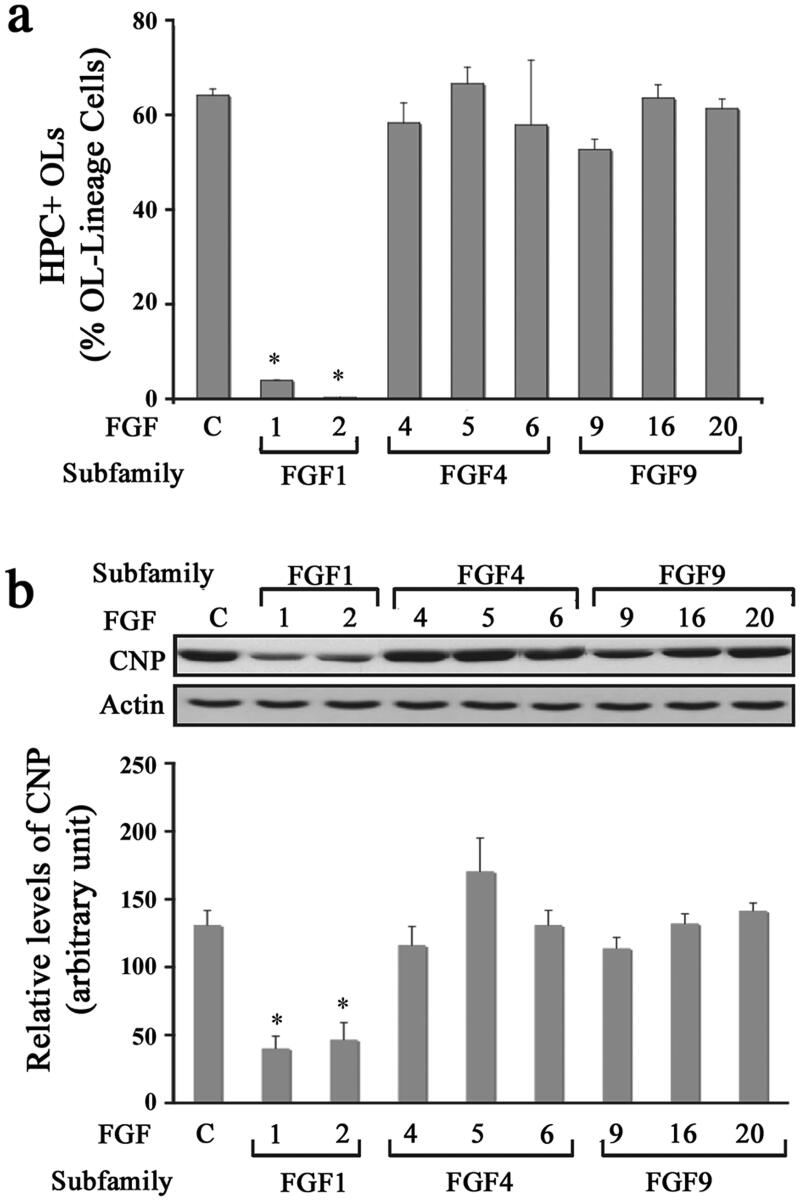
Diverse effects of FGFs on the differentiation of oligodendrocyte progenitors. OPCs were grown in the absence (control) or presence of FGF1, FGF2, FGF4, FGF5, FGF6, FGF9, FGF16, or FGF20 for 3 d and the effect of these FGFs on their differentiation was analyzed. (a) Quantification of the number of HPC + OLs expressed as percentage of total OL-lineage cells (A2B5+/O4+ cells) shows that only FGF1 and FGF2 induce a statistically significant decrease in their numbers compared with controls. (b) Quantification of the level of CNP analyzed by immunoblotting also show statistically significant decrease in its level in FGF1 and FGF2 treated cultures. Error bars represent SEM; *N* = 3 independent experiments, each performed in triplicate. *p < .05. FGF1, FGF2, FGF4, FGF6, FGF9 were used at 10 ng/ml and FGF5, FGF16, FGF20 at 20 ng/ml. Fresh doses of all FGFs were re-added on second day with a medium change. Inset, show representative immunoblots for the expression of CNP and actin as protein loading control.

In addition to enhancing the proliferation of OPCs, FGF2 is also known to suppress their terminal differentiation into OLs (Fortin et al., 2005; McKinnon et al., 1990). We therefore asked if other FGF family members could also inhibit the terminal differentiation of OPCs (Figure 2a,b). Cultures of OPCs were treated with FGF1, FGF2, FGF4, FGF5, FGF6, FGF9, FGF16, or FGF20 for 3 days and then analyzed by double immunolabeling with A2B5/O4 and HPC. We found that FGF1 and FGF2 inhibited the differentiation of OPCs compared to untreated controls, as shown by the maintenance of a progenitor-like simple morphology (data not shown) and a reduced number of HPC + OLs (Figure 2a). In contrast, FGF4, -5, -6, -9, -16, and -20 did not block the progression of OPCs into mature OLs and the cells displayed an OL-like morphology like the controls after 3 days of exposure (data not shown). To substantiate the immunofluorescence results, we performed immunoblot analysis for the differentiated OL marker CNP on cells treated with different FGFs. Quantification of signal intensity showed that only FGF1 and -2 treatment blocked the expression of CNP in differentiating OL cells (Figure 2b). We thus conclude that FGF1 and FGF2 are the only members of the FGF family that inhibit OPC differentiation.

### Effect of Different FGFs on Oligodendrocyte Progenitors in the Presence or Absence of Exogenously Added Heparin

**Figure 3.**
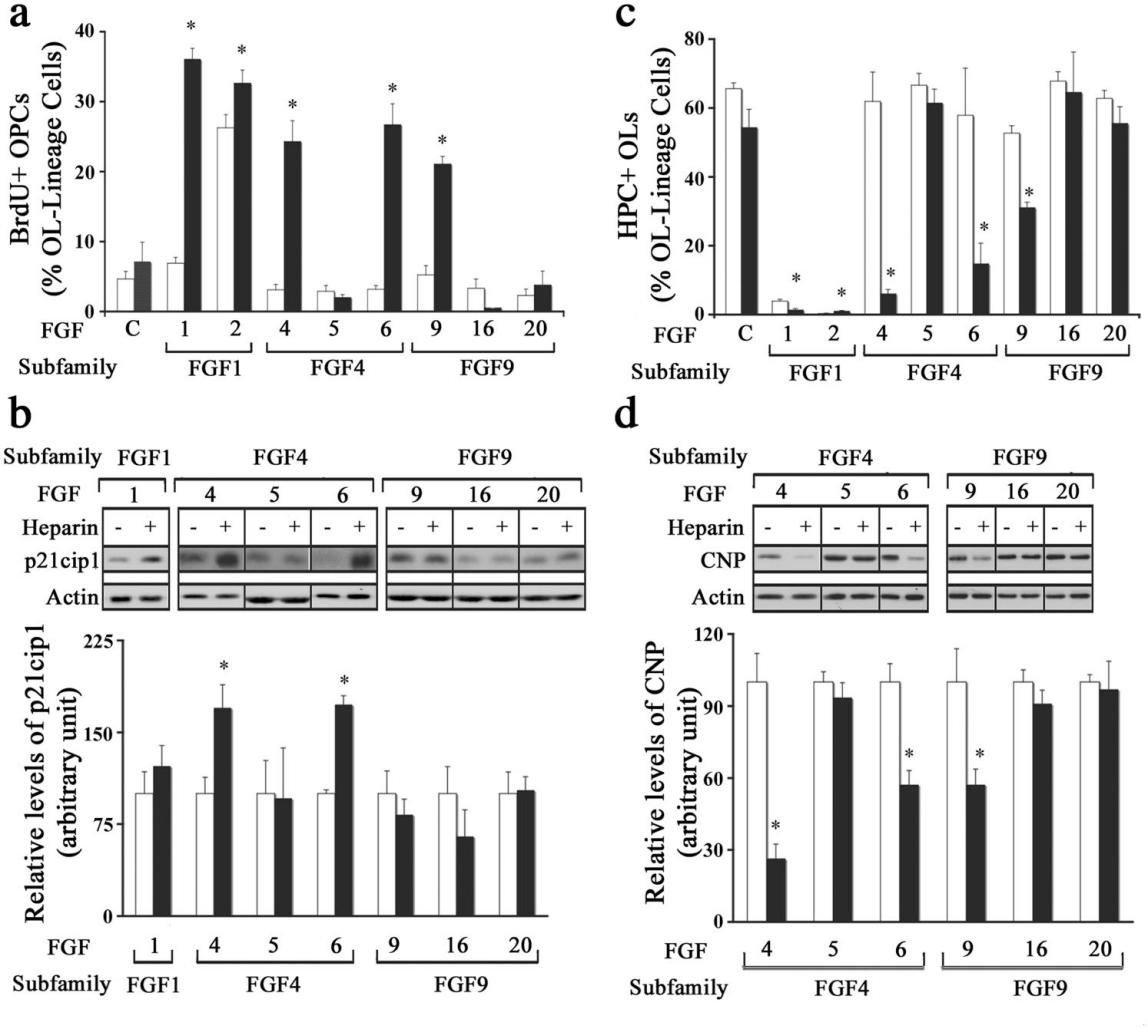
*Effect of different FGFs on oligodendrocyte progenitors in the presence or absence of exogenously added heparin.* OPCs were grown in the absence (control, open bars) or presence of heparin (black bars) with FGF1, FGF2, FGF4, FGF5, FGF6, FGF9, FGF16 or FGF20, for 1 d (a), 2 d (b,d) or 3 d (c) and analyzed by immunofluorescence microscopy or immunoblotting for their effects on OPC proliferation and differentiation. A. The quantification of the numbers of BrdU + OPCs, expressed as percentage of total OL-lineage cells (A2B5+/O4+ cells) show that exogenously added heparin significantly increases the proliferation of OPCs by FGF1, FGF2, FGF4, FGF6 and FGF9. b. Quantification of p21cip1 shows significant increase in its level by FGF4 and FGF6 treatment only in the presence of heparin. c. The quantification of the numbers of HPC + OLs, expressed as percentage of total OL-lineage cells (A2B5+/O4+ cells) show that FGF1, FGF2, FGF4, FGF6 and FGF9 significantly decreased their numbers in the presence of exogenously added heparin. d. Quantification of the level of CNP by immunoblotting shows that only FGF4, FGF6 and FGF9 significantly reduces its level in the presence of heparin. Error bars represent SEM; *N* = 3–7 independent experiments, each performed in triplicate. *p < .05. FGF1, FGF2, FGF4, FGF6, FGF9 were used at 10 ng/ml and FGF5, FGF16, FGF20 at 20 ng/ml. Fresh doses of all FGFs and heparin were re-added on second day with a medium change. Inset, show representative immunoblots for the expression of p21cip1, CNP and actin as protein loading control.

We next asked whether the observed lack of responses of OPCs to most FGFs tested was due to the limiting availability of HSPGs (as co-receptors) or whether those FGFs were totally incapable of inducing responses in OPCs. To address this question, we first examined the proliferative response of OPCs to the FGFs by measuring BrdU incorporation after treating cultures for two days with FGF1, FGF2, FGF4, FGF5, FGF6, FGF9, FGF16, or FGF20 in the absence (control) or presence of heparin (0.5 mg/ml), which satisfies the co-receptor requirement for all FGFs (Li et al., 2016). Cells were then double labeled with anti-BrdU and A2B5/O4 after 3 hr incubation with BrdU to identify proliferating OPCs. A significant increase in the number of BrdU + cells was observed in cultures co-treated with heparin and FGF1, -2, -4, -6 or -9. In contrast, the addition of heparin did not increase BrdU + OPCs numbers treated with FGF5, -16 or -20 (Figure 3a). FGF2 increased proliferation even in the absence of heparin. In parallel cultures, p21cip1 levels were evaluated by immunoblottting. We found that in the presence of HSPG while FGF4 and FGF6 showed a significant increase in p21cip1 expression, FGF1, -5, -9, -16 and -20 did not show any change (Figure 3b). Thus, while the addition of heparin was able to enhance the proliferative response of OPCs to certain FGFs, the response to other FGFs was not affected.

We next examined if heparin addition would alter the effect of different FGFs on OPC differentiation. OPC cultures were treated with FGF1, FGF2, FGF4, FGF5, FGF6, FGF9, FGF16, or FGF20 for 3 days in the absence (control) or presence of heparin (0.5 mg/ml) and then analyzed by immunolabeling with A2B5/O4 and HPC. Treatment with FGF1, -2, -4, -6 or -9 in the presence of heparin led to a significant decrease in the number of HPC + cells (Figure 3c). In contrast, the addition of heparin did not affect the number of HPC + cells in FGF5, -16 or -20-treated cultures. In parallel cultures, the level of CNP was evaluated by immunoblotting. Treatment with FGF4, -6 or -9 in the presence of heparin led to a significant reduction in CNP levels while FGF5, -16 or -20 treatment had no effect (Figure 3d). Thus, while FGF4, -6, -9 could inhibit OPC differentiation in the presence of heparin FGF5,-16,-20 did not.

Taken together, we conclude that while the limiting availability of HSPGs was apparently responsible for the inability of certain FGFs to induce responses in OPC, other FGFs (even those in the same subfamily) were found to be totally incapable of doing so.

### Effects on OPC Proliferation and Differentiation Induced by FGF4, FGF6 and FGF9 in the Presence of Heparin Were Transient

**Figure 4.**
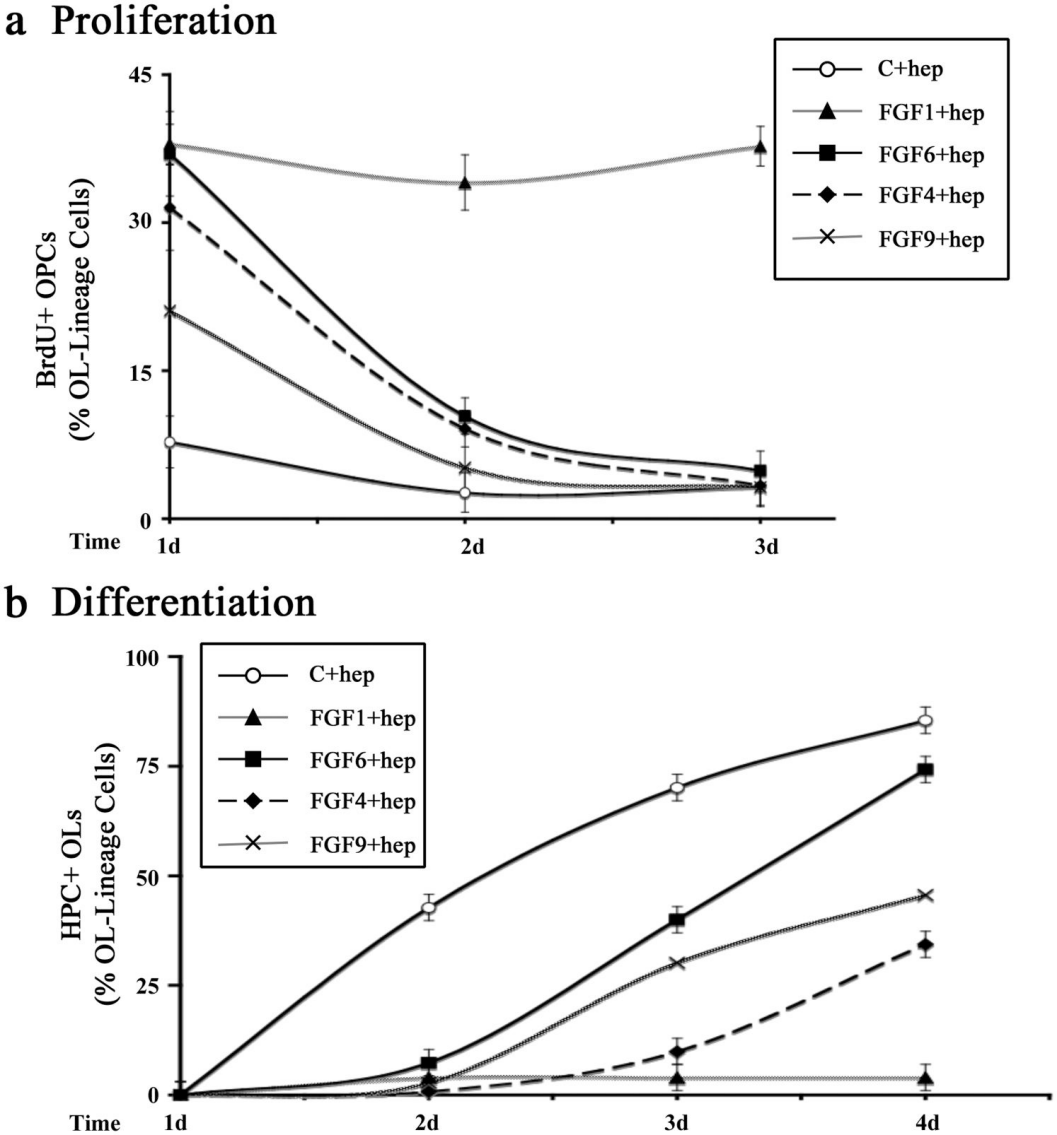
Transient effects on OPC proliferation and differentiation induced by FGF1, FGF4, FGF6 and FGF9 in the presence of heparin. OPCs were grown with FGF1, FGF4, FGF6, FGF9, or with no FGF (control) in the presence of heparin for 1-4 days and analyzed every day for their effects on OPC proliferation and differentiation. a. FGF1 treatment increased the numbers of BrdU + OPCs on day-1 which remained elevated on day-2 and -3. However, FGF4, FGF6 and FGF9 increased the numbers of BrdU + OPCs on days-1 but it was transient since it gradually reduced to control levels by day-3. b. In the presence of FGF1 differentiation of OPC to HPC + OLs was completely blocked on day-1 and remained blocked on day-2, -3 and -4. Treatment with FGF4, FGF6 and FGF9 blocked differentiation transiently on day-1 and -2 but the numbers of HPC + OLs gradually increased on day-3 and -4. Error bars represent SEM; *N* = 3–7 independent experiments, each performed in triplicate. *p < .05. FGF1, FGF2, FGF4, FGF6, FGF9 were used at 10 ng/ml and FGF5, FGF16, FGF20 at 20 ng/ml. Fresh doses of all FGFs and heparin were re-added on second day with a medium change.

In our previous studies we have found that FGF2 mediates a sustained increase of OPC proliferation (McKinnon et al., 1990; Fortin et al., 2005). To determine whether in the presence of heparin other FGFs induce a similar sustained increases in OPC proliferation, we examined FGF1, -4, -6 or -9 induced proliferation of OPCs as a function of time by immunolabelling with anti-BrdU and A2B5/O4 following BrdU exposure (Figure 4a). Quantification of the number of BrdU + OPCs at days-1, -2 and -3 after FGF addition showed that FGF1 treatment in the presence of heparin caused a sustained increase of OPC proliferation. However, only a transient increase of 1–2 d was observed following FGF4, -6 or -9 treatment. Specifically, treatment of OPCs with FGF4 or -6 in the presence of heparin led to BrdU + incorporation at rates similar to that of FGF1 on day-1 that drastically fell on day-2 which fell to control levels by day-3 (Figure 4a). The observed FGF9 mediated increase was less pronounced than FGF1, -4, -6 on day-1 but it also fell to control levels by day-3. We conclude that unlike the sustained induction of OPC proliferation by FGF1 and -2, the other FGFs, FGF4, -6, -9 only induced a transient increase which could not be maintained over time, even in the presence of heparin.

In our previous studies we found that in the presence of FGF2, OPCs do not differentiate into OLs and continue to remain as OPCs over long periods of time. To investigate if the heparin-mediated inhibition of OPC differentiation by FGF4, -6, -9 was sustained or transient, we examined the time course of OPC differentiation in the absence (control) or presence of FGF4, -6, or -9 (plus heparin) by immunolabeling cells with A2B5/O4 and HPC (Figure 4b). We found that in untreated controls, OPCs began to differentiate into OLs expressing HPC at day-2 in culture and that the number of differentiated OLs increased in number reaching approximately 85% by day-4. In the presence of FGF1 (plus heparin) OPC differentiation remained arrested throughout the 4 days of treatment. However, OPCs treated with FGF6 or -9 (plus heparin) began to differentiate on day-3 and by day-4, 74% and 46% of these OPCs had completely differentiated into HPC + OLs. Although the rate of differentiation was somewhat slower following FGF4 (plus heparin) treatment, by day-4 34% of OPCs had differentiated into HPC + OLs. We conclude that unlike the sustained inhibition of OPC differentiation by FGF1 in the presence of heparin and FGF2 without heparin (as noted previously), inhibition by FGF4, -6, and -9 was transient and could not be maintained over time, even in the presence of heparin.

### Differential Effects of FGFs on Mature Oligodendrocytes

We have previously shown that exposing mature OLs in culture to FGF2 resulted in multiple responses, including the (1) down-regulation of major myelin proteins and FGFR2, and (2) cell cycle reentry (Bansal & Pfeiffer, 1997). Here we investigated the responses of mature OLs to other FGF family members of interest.

**Figure 5.**
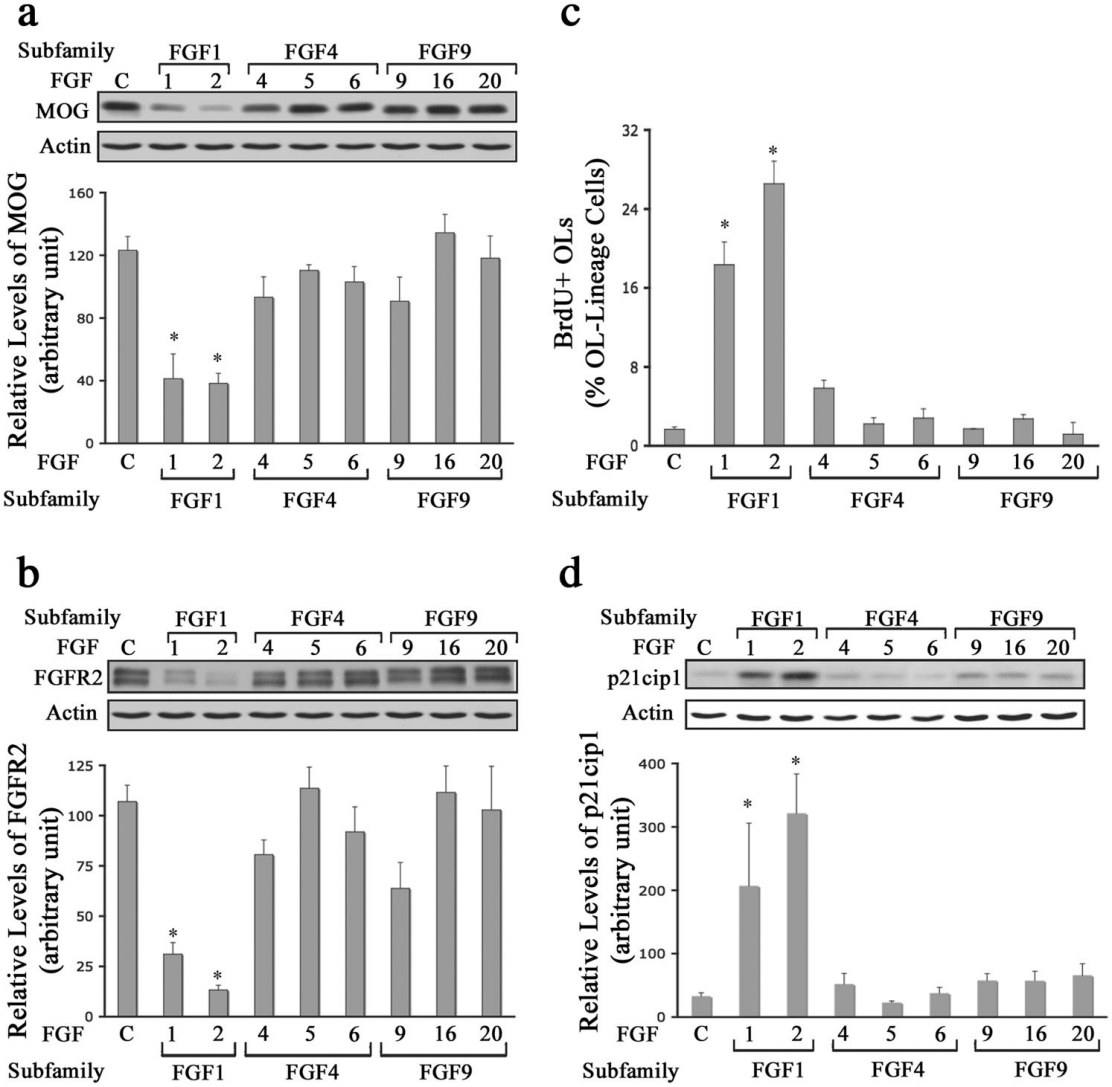
Differential effects of FGFs on mature oligodendrocyte. Mature OLs were grown in the absence (control) or presence of FGF1, FGF2, FGF4, FGF5, FGF6, FGF9, FGF16, or FGF20 for 2 d, and analyzed for the expression of myelin proteins MOG, FGFR2, BrdU incorporation and p21cip1 expression. a,b. Quantification of the levels of MOG and FGFR2 by immunoblotting show that only FGF1 and FGF2 induce a statistically significant downregulation of these proteins compared to controls. c,d. Increase in the numbers of BrdU + OLs and levels of p21cip1 also show that only FGF1 and FGF2 treatment induces mature OLs to re-enter the cell cycle. Error bars represent SEM; *N* = 3–7 independent experiments, each performed in triplicate. *p < .05. FGF1, FGF2, FGF4, FGF6, FGF9 were used at 10 ng/ml and FGF5, FGF16, FGF20 at 20 ng/ml. Inset, show representative immunoblots for the expression of MOG, FGFR2, p21cip1 and actin as protein loading control.

OPCs were grown in culture until they differentiated into mature OLs. We then exposed them to FGF1, FGF2, FGF4, FGF5, FGF6, FGF9, FGF16, or FGF20 for 2 d and evaluated the expression of myelin proteins (MOG and FGFR2) by immunoblotting (Figure 5a,b). Consistent with our previous observation (Bansal & Pfeiffer, 1997), FGF2 exposure led to a down-regulation of several myelin proteins including, PLP, CNP, NF155, MBP and MOG, (as an example only MOG is shown) and FGFR2 [FGF2 reduced MOG and FGFR2 expression to 38 ± 6% and 13 ± 2% of control, respectively (Figure 5a,b)]. FGF1 was the only other FGF to induce statistically significant reductions in MOG and FGFR2 levels. In contrast, FGF4, -5, -6, -9, -16 or -20 did not induce significant down-regulation of MOG or FGFR2 compared to control. We conclude that FGF1 and FGF2 are the only members of the FGF family that down-regulate the expression of major myelin proteins in mature OLs.

FGF-mediated re-entry of differentiated OLs into the cell cycle was analyzed by immunolabeling of mature OLs with O4 and anti-BrdU (after 3 h exposure of cells to BrdU) and by immunoblot analysis for p21cip1, following treatment with FGF1, FGF2, FGF4, FGF5, FGF6, FGF9, FGF16, or FGF20 for 2 d (Figure 5c,d). Whereas differentiated OLs rarely incorporated BrdU (<3%), the application of FGF1 or FGF2 increased the total number of OLs incorporating BrdU by more than 6-fold (Figure 5c). Furthermore, p21cip1 levels quantified by immunoblotting also demonstrated a statistically significant increase following FGF1 and FGF2 treatment (Figure 5d). In contrast, exposure of mature OLs to FGF4, -5, -6, -9, -16 or -20 showed statistically insignificant effects on both the number of BrdU + cells and on the levels of p21cip1 in comparison to the control. We conclude that FGF1 and FGF2 are the only members of the FGF family that trigger OLs to re-enter the cell cycle.

### Effect of Different FGFs on Mature Oligodendrocytes after the Addition of Exogenous Heparin

**Figure 6.**
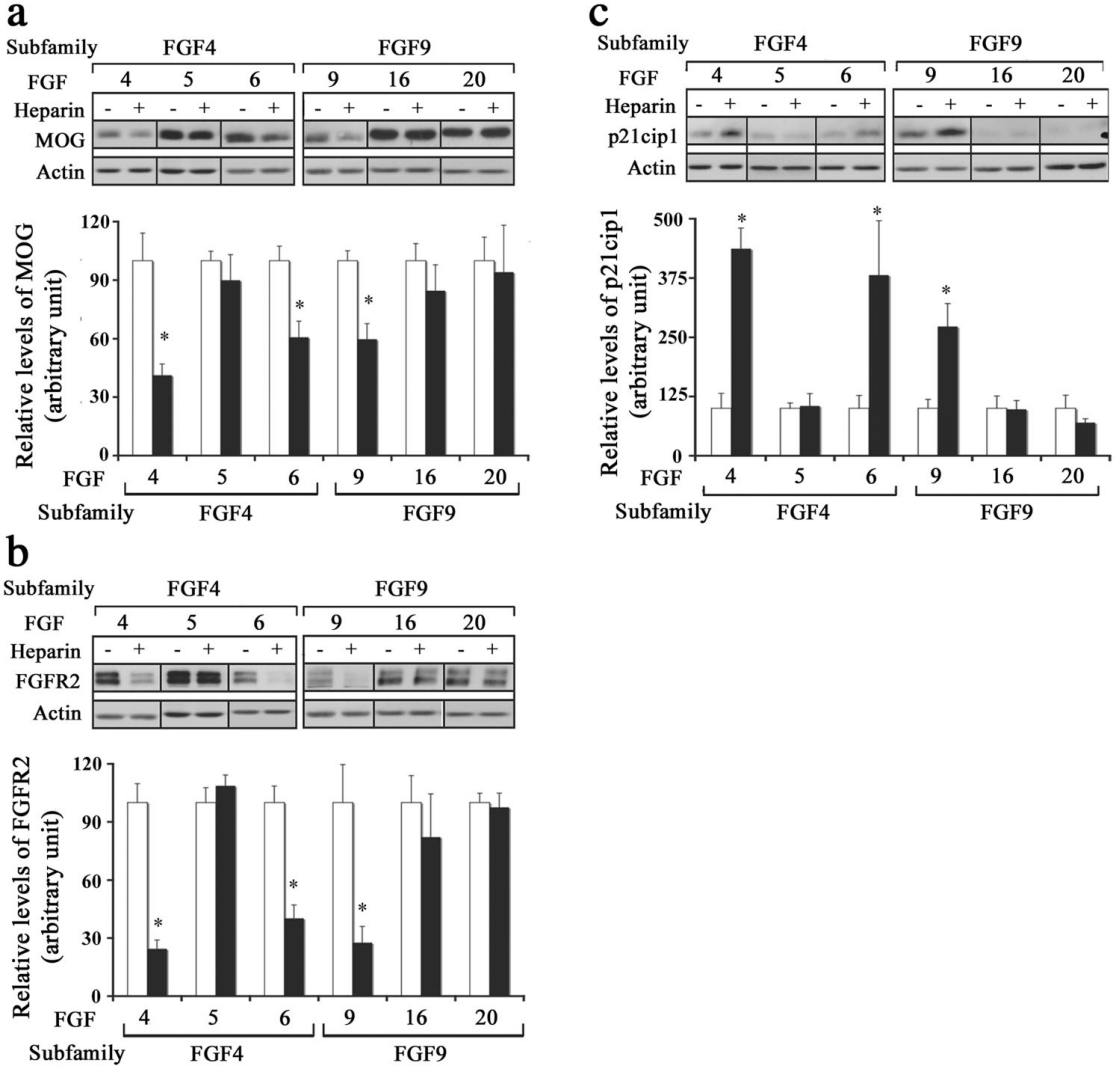
Effect of different FGFs on mature oligodendrocytes after the addition of exogenous heparin. Mature OLs were grown in the absence (control, open bars) or presence of heparin (black bars) and treated with FGF4, FGF5, FGF6, FGF9, FGF16 or FGF20 for 2 d and analyzed for myelin protein expression and cell cycle re-entry. (a,b) immunoblot analysis and quantification of myelin proteins (MOG, FGFR2) show that FGF4, FGF6, and FGF9 significantly downregulated these proteins in the presence of heparin. c, Quantification of p21cip1 show that in the presence of heparin FGF4, FGF6, and FGF9 cause a significant increase in its levels. Error bars represent SEM; *N* = 3–4 independent experiments, each performed in triplicate. *p < .05. FGF1, FGF2, FGF4, FGF6, FGF9 were used at 10 ng/ml and FGF5, FGF16, FGF20 at 20 ng/ml. Inset, show representative immunoblots for the expression of MOG, FGFR2, p21cip1 and actin as protein loading control.

We next asked whether the inability of mature OLs to downregulate their myelin proteins after exposure to certain FGFs was due to the limiting availability of endogenous HSPGs. To address this question, we exposed mature OLs to FGF1, FGF2, FGF4, FGF5, FGF6, FGF9, FGF16, or FGF20 in the absence (control) or presence of heparin (0.5 mg/ml) for 2 d and analyzed them by immunoblotting for MOG, FGFR2. We found that the addition of heparin led to a significant downregulation of the levels of MOG and FGFR2 in differentiated OLs treated with FGF4, -6 or -9 (Figure 6a,b). However, the addition of heparin did not lead to a significant difference in the levels of MOG or FGFR2 in OLs treated with FGF -5, -16, or -20. We conclude, that in the presence of heparin, while FGF4, -6, -9 were able to downregulate myelin protein expression, FGF5, -16, -20 were unable to do so.

The re-entry of differentiated OLs into the cell cycle by exposure to different FGFs in the presence of heparin was analyzed by immunoblotting for p21cip1. We found that the addition of heparin led to significant increases in the level of p21cip1 in mature OLs treated with FGF4, -6 or -9, but not with FGF5, -16 or -20 (Figure 6c). Taken together, we conclude that while the limiting availability of HSPGs was apparently responsible for the inability of FGF4, -6, -9 to induce responses in mature OLs, FGF5, -16 and -20 were incapable of doing so.

## Discussion

**Figure 7.**
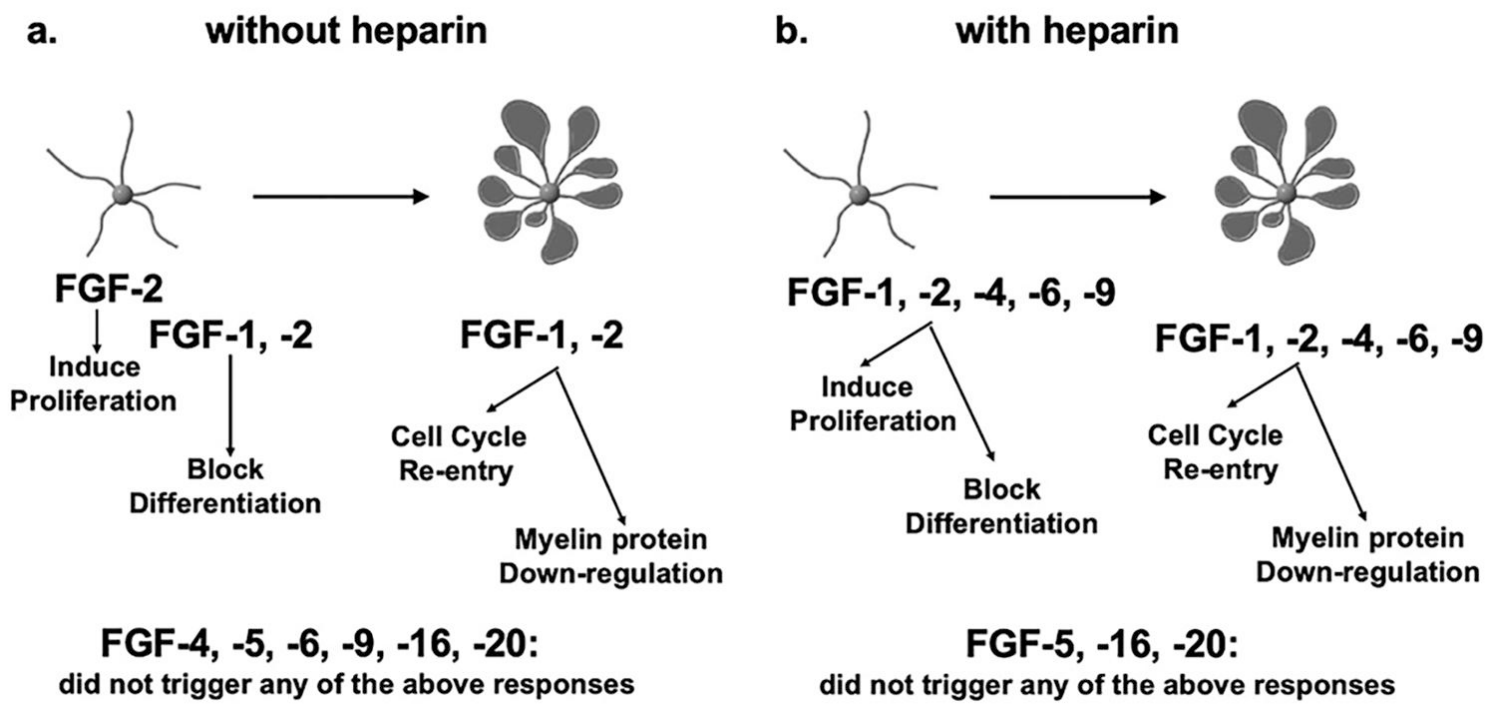
Summary of responses of OL-lineage cells to different FGFs. OPCs (represented here by cell with simple morphology) and mature OL cells (represented here by complex cells with prominent membranes) were cultured in the absence (a) or presence (b) of heparin. The effects of members of the *FGF1 (FGF1, FGF2), FGF4 (FGF4, FGF5, FGF6) and FGF9 (FGF9, FGF16, FGF20)* were examined on proliferation and differentiation of OPCs, and on cell cycle re-entry and down-regulation of myelin proteins by mature OLs. FGF2 induced all responses strongly but responses to FGF4,-6,-9 were transient and only observed in the presence of heparin. Neither OPCs nor OLs exhibited any responses to FGF5,-16,-20 even in the presence of heparin or at higher concentrations. Note that structurally similar FGFs within subfamilies did not always show similarities in their biological effects on OL-lineage cells.

Oligodendrocyte development and function are modulated by an array of signaling molecules, their receptors and subsequent activation of intracellular signaling pathways. Using our enriched oligodendrocyte cultures, we previously showed that FGF2 is a potent mitogen for cultured OPCs and inhibits their terminal differentiation. We further showed that differentiated mature OLs exposed to FGF2 downregulate their myelin proteins and FGFR2 and reenter the cell cycle (Bansal & Pfeiffer, 1997; Fortin et al., 2005). Finally, we reported that FGF8 family members (FGF8, -17, -18) elicited only a subset of these responses (Fortin et al., 2005). These findings led us to examine the effects of the remaining FGF subfamilies to obtain a comprehensive knowledge of the roles of FGFs on OL-lineage cells (Figure 7).

Several FGFs from subfamilies 1, 4 and 9 triggered responses in OL-lineage cells that differed from responses to FGF2 in important ways. While cellular responses to FGF -4, -6 and -9 were qualitatively similar to responses to FGF-2, these FGFs could induce these responses only when heparin was added, suggesting that the co-receptor requirement of these FGFs is distinct from that of FGF2. Heparin may have enhanced the bioavailability of FGF4, -6 and -9 and intensified the strength of their effect. Thus, these FGFs are different from FGF2 not in terms of the nature of the responses they are able to elicit but in terms of their potency. Furthermore, unlike FGF2, the effects of FGF-4, -6 and -9 were transient even in the presence of heparin. It is unlikely that this was due to their differential stabilities because fresh FGF doses were added every second day during the experiment. Thus, these FGFs are also different from FGF2 in terms of the duration of their action. Finally, responses to FGF5, -16, -20 diverged completely, as OL-lineage cells did not respond to these FGFs at all even at higher concentrations or after the addition of heparin. Given the diversity observed in the regulation of OL-lineage cells by these FGF family members, it is possible that they may play important roles in normal OL development and myelination, while FGF2, whose responses are not so selective, may be associated with OL/myelin pathology (Bansal, 2002). Consistent with this notion, FGF2 expression is increased in demyelinated multiple sclerosis lesions (Clemente et al., 2011) and FGF2 null mice treated with cuprizone to induce demyelination showed enhanced remyelination relative to control mice (Messersmith et al., 2000) suggesting that upregulated FGF2 expression was detrimental for OL/myelin. Thus, the upregulated FGF2 expression in macrophages and astrocytes seen in demyelinating lesions, although clinically relevant, may not be a true reflection of its normal function during OL development and myelination.

HSPGs such as heparin are known to bridge ligand-receptor interactions in multiple signaling systems (Li et al., 2016; Xie & Li, 2019) and mechanistically, the formation of FGF ternary complexes with HSPG (co-receptors) and with FGFRs increases the FGF-FGFR affinity and potency (Ibrahimi et al., 2004). We have previously shown that HSPGs namely, syndecan-2 and -4 and glypican mRNA and protein are expressed by OPCs and mature OL in a developmentally regulated manner (Bansal et al., 1996b; Winkler et al., 2002). Thus, developmental expression of specific HSPGs in OL-lineage cells or in neighboring cells can control the accessibility of FGFs to their receptors in ways that can modulate OPC function. Interestingly, glucuronyl C5-epimerase ablation with concomitant loss in L-iduronic HSPG epitopes alters responses to FGF2 but not to FGF10, which disrupts skeletal and kidney formation and causes postnatal death (Jia et al., 2009). This suggests that HSPG epitopes interact only with some FGF ligands that signal specific events in development, providing another level of selective regulation by different FGFs.

In addition to the role played by co-receptors (HSPGs) in determining how different FGF family members may regulate the responses of OL-lineage cells, it is likely that FGF-FGF-receptor interactions may also govern their responses. Selectivity of different FGFs to activate different FGF receptors has been shown in cell lines expressing a single recombinant receptor (Itoh & Ornitz, 2004; Zhang et al., 2006). We have shown that the expression of FGFR-1, -2 and -3 is temporally regulated in cultured OL-lineage cells (Bansal et al., 1996a; Fortin et al., 2005). Specifically, FGFR1 is expressed in OPCs and mature OLs, FGFR2 is present in mature OLs (at much greater levels than FGFR1) and FGFR3 is expressed in OPCs and downregulated in mature OLs. Thus, the interaction of different FGFs with the developmentally regulated FGF receptors, together with the developmentally regulated HSPGs can potentially induce multiple responses in the OL-lineage cells.

Members of FGF subfamilies, grouped according to sequence similarities, are assumed to trigger similar responses. However, our results here indicate that members in the same subfamily can trigger diverse responses in OL-lineage cells. For example, FGF9 elicited responses in OL-lineage cells but FGF16 and -20, members of the same FGF subfamily, failed to do so, even at a higher concentration and in the presence of added heparin. Similarly, unlike FGF4 and -6, FGF5 (a member of the same subfamily) could not induce any responses. This suggests that the sequence similarity among FGF subfamily members may not be a perfect predictor of their biological responses.

Selectivity of different FGFs for different FGFRs is an emerging area of investigation (Karl et al., 2020). FGF receptor dimer stability may determine mitogenic versus metabolic functions of FGFs (Huang et al., 2017). Thus, it is possible that some FGFs within subfamilies may favor the formation of distinct receptor complexes (dimers vs oligomers) in OL-lineage cells. Further, this may be modulated by the participation of certain HSPGs. Subsequent recruitment of downstream intracellular signaling molecules by the FGF-FGFR-HSPG complex (Brewer et al., 2015) such as the adapter protein Frs2 and MAPK, AKT, mTOR may further provide diversity in the responses of cells. We have shown that in cultured OLs, treatment with FGF2 leads to robust phosphorylation of Frs2, MAPK and Akt (Bryant et al., 2009) and in transgenic mice these signaling molecules function downstream of FGFR in the OL-lineage cells (Furusho et al., 2017, 2020; Guardiola-Diaz et al., 2012).

In summary, our study revealed that FGFs from subfamilies 1, 4 and 9 showed diversity in their regulation of OL-lineage cells. Furthermore, the structure-based classification of FGF subfamilies is a productive organizing framework for understanding FGFs but with respect to OL development, it does not capture the diversity of responses that are possible or the wide variety of developmental outcomes that can result from a system that contains such inherent combinatorial potential. Finally, this study further emphasizes the selectivity and importance of HSPG molecules as FGF co-receptors for the OL-lineage cells.
